# Association between blood lipid levels and the risk of liver cancer: a systematic review and meta-analysis

**DOI:** 10.1007/s10552-024-01853-9

**Published:** 2024-02-20

**Authors:** Zhihui Zhang, Shicong Xu, Meixuan Song, Weirong Huang, Manlin Yan, Xianrong Li

**Affiliations:** 1 School of Nursing, Southwest Medical University, Luzhou, 646000 China; 2 Department of Gastrointestinal surgery, The Affiliated Hospital, Southwest Medical University, Luzhou, 646000 China

**Keywords:** Liver neoplasms, Blood lipids, Cohort study, Meta-analysis

## Abstract

**Purpose:**

The association between blood lipid levels and the risk of developing liver cancer remains a subject of ongoing debate. To elucidate this association, we conducted a meta-analysis by systematically incorporating data from all relevant prospective cohort studies.

**Methods:**

We conducted a systematic search of the PubMed, Embase, Web of Science, and Cochrane Library databases covering studies published from database inception through July 2023. This study included prospective cohort studies related to lipid profiles (e.g., total cholesterol (TC), triglyceride (TG), high-density lipoprotein cholesterol (HDL-C), and low-density lipoprotein cholesterol (LDL-C) levels) that reported hazard ratios (HRs) or relative risks (RRs) with corresponding 95% confidence intervals (95% CIs) to investigate their association with the risk of liver cancer. During the analysis process, we used fixed-effects or random-effects models based on the level of heterogeneity among the studies and obtained pooled risk ratios using these models. To ensure the robustness and reliability of the study findings, we also conducted sensitivity analyses and publication bias analyses.

**Results:**

After conducting a systematic search, 12 studies were identified from a total of 11,904 articles and were included in the meta-analysis. These studies included a combined population of 10,765,221 participants, among whom 31,055 cases of liver cancer were reported. The analysis revealed that the pooled HR for the serum TC concentration (highest versus lowest) was 0.45 (95% CI = 0.35–0.58, I^2^ = 78%). For TGs, the HR was 0.67 (95% CI = 0.46–0.96, I^2^ = 86%), while for HDL-C, the HR was 0.72 (95% CI = 0.58–0.90, I^2^ = 65%). The HR for LDL-C was 0.51 (95% CI = 0.23–1.13, I^2^ = 93%).

**Conclusion:**

The findings of this study indicate that serum TC, TG, and HDL-C levels are negatively associated with liver cancer risk, suggesting that higher concentrations of these lipids are associated with a reduced risk of liver cancer. However, no significant association has been found between LDL-C levels and liver cancer risk.

**Supplementary Information:**

The online version contains supplementary material available at 10.1007/s10552-024-01853-9.

## Introduction

Primary liver cancer refers to malignant tumors originating from liver cells. The main types of primary liver cancer include hepatocellular carcinoma (HCC) and intrahepatic cholangiocarcinoma [1]. Globally, primary liver cancer ranks as the sixth most common cancer and the third deadliest cancer [2, 3]. According to data from the World Health Organization (WHO), approximately 8,00,000 people are diagnosed with primary liver cancer globally each year [4]. The incidence of liver cancer is particularly high in Asia, with China having the highest incidence, accounting for approximately half of all global cases [5]. The main risk factors for liver cancer are chronic hepatitis B virus (HBV), hepatitis C virus (HCV), and fatty liver diseases [6, 7]. Several studies have suggested that abnormalities in blood lipid levels may be associated with an increased risk of liver cancer [8, 9]. Dyslipidemia may lead to the accumulation of fat in the liver and chronic inflammatory responses, thereby promoting the development of liver cancer [10].

Blood lipids refer to lipid or fat substances in the blood. Lipids are a class of biomolecules that mainly include total cholesterol (TC), triglycerides (TGs), high-density lipoprotein cholesterol (HDL-C), and low-density lipoprotein cholesterol (LDL-C) [11, 12]. Blood lipids play essential physiological roles in the body, such as providing energy, constructing cell membranes, synthesizing hormones, and regulating other biological processes [13]. The link between blood lipid levels and cardiovascular diseases has been well established [14, 15], and associations of blood lipid levels with colorectal cancer [16], prostate cancer [17], and breast cancer [18] have also been firmly established. Moreover, blood lipid levels are associated with the prognosis of various cancers [19–21].

The association between blood lipid levels and the risk of liver cancer is a topic of considerable research interest. However, there is currently some controversy regarding this association. Some studies suggest that dyslipidemia may be associated with an increased risk of liver cancer [22, 23], while others report no clear association [24]. These discrepancies may be attributed to the complex interplay between dyslipidemia and other risk factors, such as obesity, diabetes, and alcohol consumption, collectively influencing the risk of liver cancer. To assess the association between blood lipid levels and the risk of liver cancer, a meta-analysis including all prospective studies relevant to blood lipid components was conducted. By employing systematic review and meta-analysis methods, we aimed to explore the association between blood lipid levels and the risk of liver cancer, with the hope of providing further insights into this unclear association.

## Materials and methods

This study strictly adhered to the guidelines of the Preferred Reporting Items for Systematic Reviews and Meta-Analyses (PRISMA) statement [25] (Supplementary Table S1) and has been registered in PROSPERO (CRD42023442073).

### Inclusion criteria

The inclusion criteria were as follows: [1] cohort studies; [2] studies involving adult participants (aged ≥ 18 years); [3] studies clearly stating liver cancer as the outcome of interest; [4] studies providing explicit reports on the effect size of serum TC, TG, HDL-C, or LDL-C levels; and [5] studies reporting effect size measurements as hazard ratios (HRs) or relative risks (RRs) with corresponding 95% confidence intervals (95% CIs).

### Exclusion criteria

The exclusion criteria were as follows: [1] study design: studies with small sample sizes or significant selection bias; [2] age range: studies that did not cover the age range of the target participants; [3] outcome clarity: studies that did not explicitly state the study outcome or relevant indicators; [4] missing data: studies with substantial missing data or for which valid data could not be obtained; and [5] duplicate publications: studies that had been previously published in other literature.

### Search strategy

A combination of subject terms and free-text words was used, and all the search terms were used in both singular and plural forms. Two researchers independently searched the Embase, PubMed, Web of Science, and Cochrane Library databases to identify studies investigating the association between blood lipid levels and liver cancer risk. The search was limited from database inception up to July 2023. Our search terms were as follows: serum lipids, total cholesterol, TC, triglycerides, TG, high-density lipoprotein, HDL-C, low-density lipoprotein, LDL-C, and liver cancer. To ensure the inclusion of all relevant studies, the researchers also manually searched other relevant journal articles, reviews, and sources and thoroughly reviewed the reference lists of the included studies. When multiple articles from the same cohort were published, we prioritized the study with the longest and most recent follow-up duration.

### Data extraction

We extracted the following information from each study: the first author’s name, publication year, study location, sample size, duration of follow-up, and HR or RR and the corresponding 95% CI between the highest and lowest serum concentrations. Two researchers (ZHZ and SCX) independently extracted the data from the eligible studies. In cases of disagreement, the entire team resolved the issue through consensus. When multiple risk estimates were reported in a single study, we selected the estimate that had the most comprehensive adjustment for confounding factors.

### Quality assessment

The quality assessment of each study was independently conducted by two researchers (MXS and WRH) using the Newcastle–Ottawa Scale (NOS) for quality assessment [26]. Based on the NOS scores, the quality of each study was categorized into three groups: low quality (< 5 points), medium quality (5–7 points), and high quality (≥ 8 points).

### Statistical analysis

Data analysis was conducted using Review Manager software (version 5.4.1) and Stata software (version 17.0). We extracted data on risk estimates from each study and calculated HRs and the corresponding 95% CIs using either fixed-effects or random-effects models based on the degree of heterogeneity observed. Heterogeneity was assessed using the I^2^ statistic, where I^2^ > 50% indicated significant heterogeneity, I^2^ values between 30% and 50% indicated moderate heterogeneity, and I^2^ < 30% suggested the absence of significant heterogeneity. If I^2^ was < 50%, we used the fixed-effects model; otherwise, the random-effects model was applied. Additionally, we employed the meta-regression method to assess the variability between subgroups and explored the potential for publication bias through the symmetry of funnel plots and the Begg and Egger tests. A p-value less than 0.05 indicated statistical significance, and to evaluate the stability of the results, sensitivity analysis was performed.

## Results

### Study characteristics

**Fig. 1:**
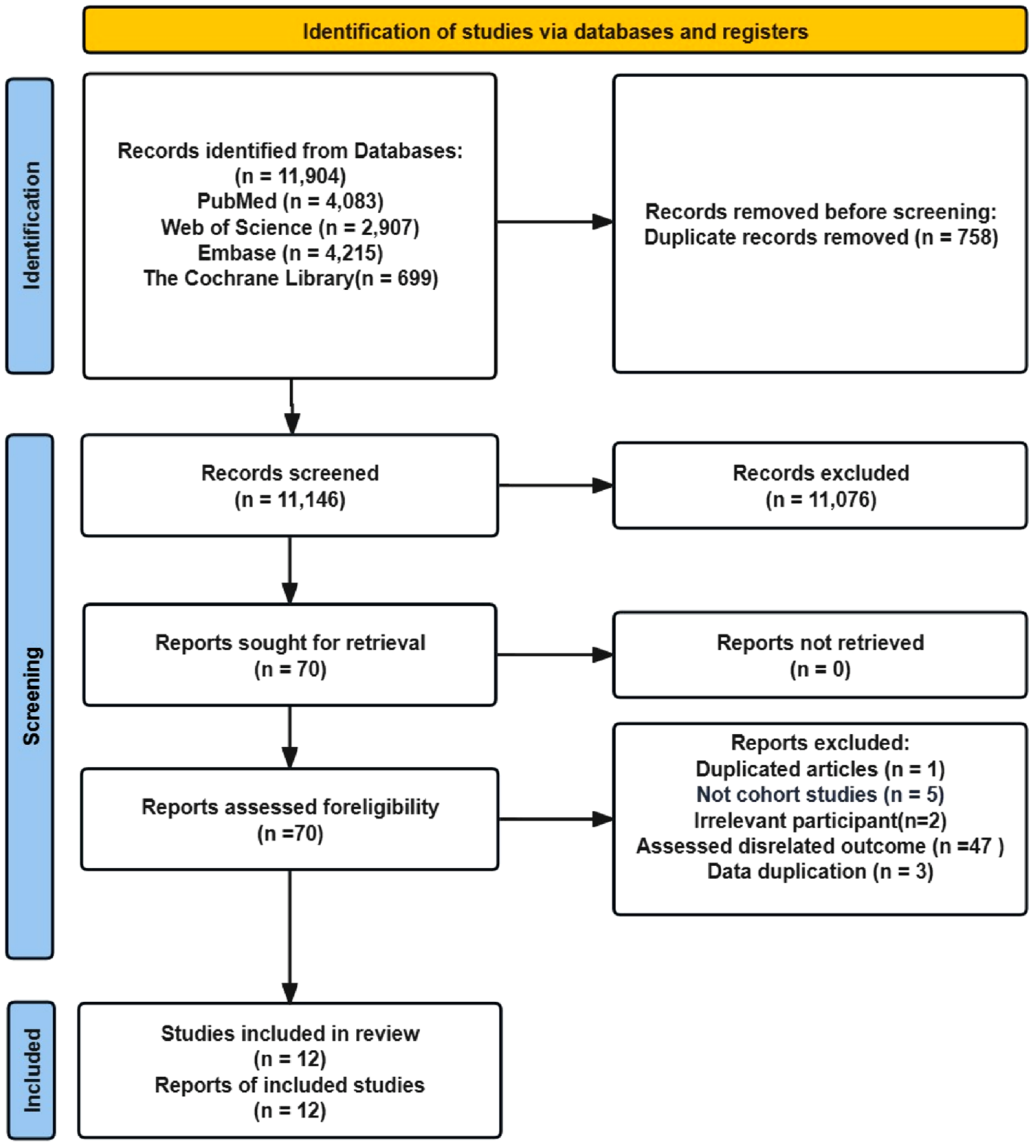
Flowchart of the literature selection process

After conducting a systematic search, a total of 11,904 articles were identified. After removing 758 duplicates, the titles and abstracts of the remaining 11,146 articles were reviewed, leading to the selection of 70 articles for full-text screening. After excluding studies unrelated to blood lipid levels and liver cancer risk, a total of 12 articles were included in the meta-analysis (Fig. 1**)**, involving 10,765,221 participants, among whom 31,055 cases of liver cancer were reported. These studies were published between 2009 and 2022; 8 were from Asia [27–34], and 4 were from Europe [35–38]. The NOS scores of the included studies ranged from 7 to 9, with 5 studies obtaining a score of 7 and 7 studies obtaining a score of 8 to 9 (Supplementary Table S3).

### Meta-analysis

#### Serum total cholesterol

**Fig. 2:**
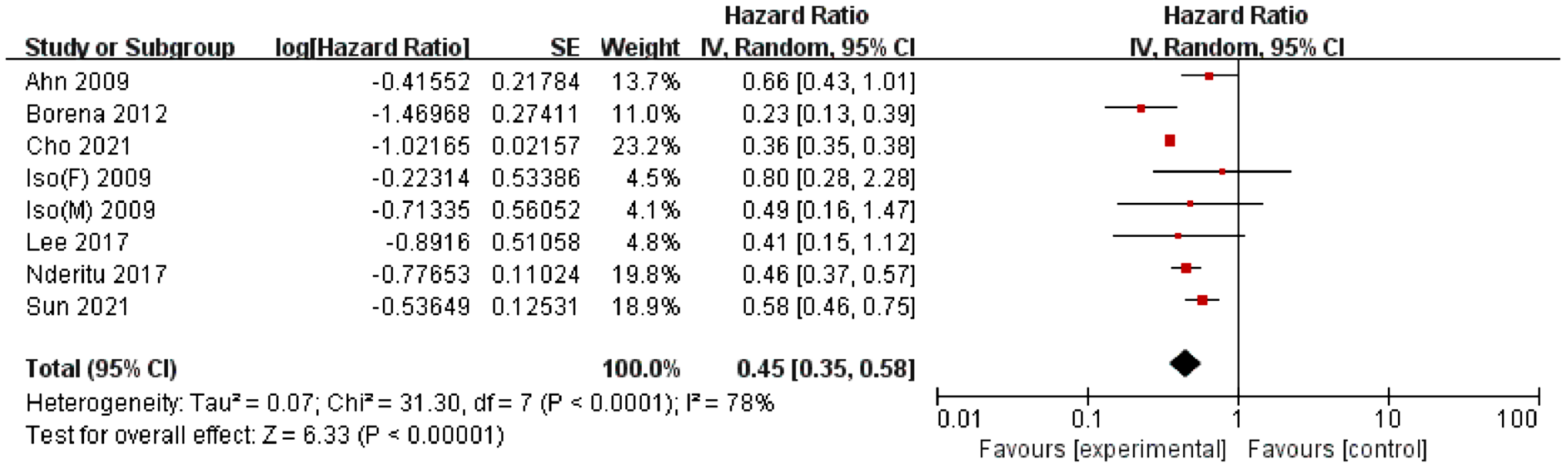
Forest plot of the association between total cholesterol and liver cancer risk

A total of 7 studies, published between 2009 and 2021, reported the association between serum TC and liver cancer; these studies involved 9,812,439 participants, among whom 28,586 cases of liver cancer were reported. Among these studies, 4 were conducted in Asia [28, 30, 31, 34] and 3 were conducted in Europe [35–37]. A meta-analysis of the 7 studies demonstrated a significant negative association between serum TC and liver cancer risk (HR = 0.45, 95% CI = 0.35–0.58; *P* < 0.01). Significant heterogeneity was detected (I^2^ = 78%, *P* < 0.01) (Fig. 2). Egger’s test (*P* = 0.14) and Begg’s test (*P* = 1.00) did not provide evidence of publication bias. Furthermore, visual inspection of the funnel plot did not reveal any asymmetry (Supplementary Fig. S5).

### Serum triglyceride

**Fig. 3:**
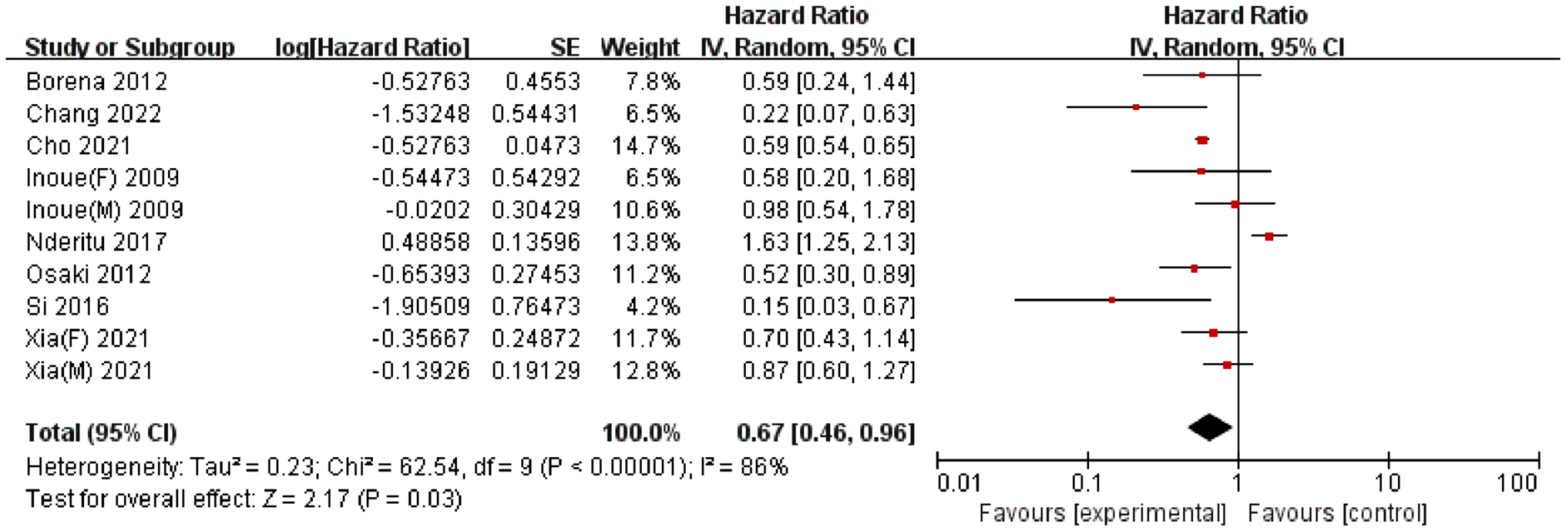
Forest plot of the association between triglyceride levels and liver cancer risk

A total of 8 studies, published between 2009 and 2022, reported the association between serum TG levels and liver cancer; these studies involved 10,199,390 participants, among whom 28,706 cases of liver cancer were reported. Among these studies, 5 were conducted in Asia [27–29, 32, 33] and 3 were conducted in Europe [35, 36, 38]. A meta-analysis of the 8 studies demonstrated a significant negative association between serum TG levels and liver cancer risk (HR = 0.67, 95% CI = 0.46–0.96, *P* = 0.03) (Fig. 3). Significant heterogeneity was detected (I^2^ = 86%, *P* < 0.01). Egger’s test (*P* = 0.79) and Begg’s test (*P* = 0.21) did not provide evidence of publication bias. Furthermore, visual inspection of the funnel plot did not reveal any asymmetry (Supplementary Figure S5).

### Serum high-density lipoprotein cholesterol

**Fig. 4:**
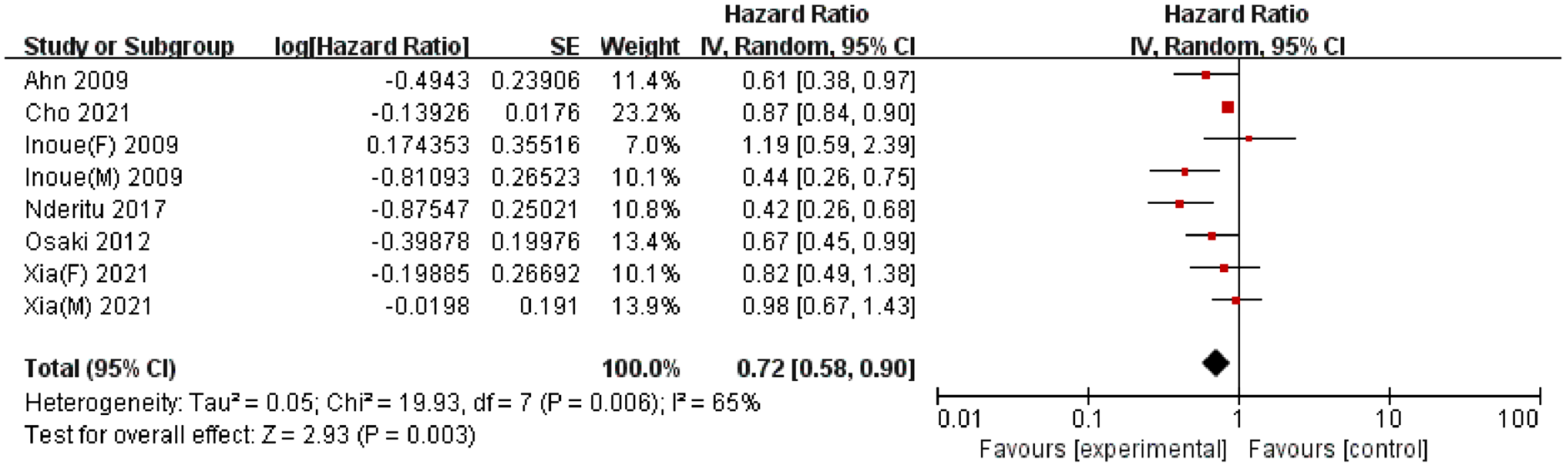
Forest plot of the association between high-density lipoprotein cholesterol levels and liver cancer risk

A total of 6 studies, published between 2009 and 2021, reported the association between serum HDL-C levels and liver cancer risk; these included 9,593,597 participants, among whom 28,367 cases of liver cancer were reported. Among these studies, 3 were conducted in Asia [28, 29, 32] and 3 were conducted in Europe [35, 37, 38]. A meta-analysis of the 6 studies demonstrated a significant negative association between the serum HDL-C concentration and liver cancer risk (HR = 0.72, 95% CI = 0.58–0.90, *P* < 0.01). Significant heterogeneity was detected (I^2^ = 65%, *P* < 0.01) (Fig. 4). Egger’s test (*P* = 0.13) and Begg’s test (*P* = 0.54) did not provide evidence of publication bias. Furthermore, visual inspection of the funnel plot did not reveal any asymmetry (Supplementary Fig S5).

### Serum low-density lipoprotein cholesterol

**Fig. 5:**
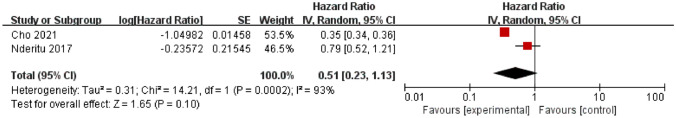
Forest plot of the association between low-density lipoprotein cholesterol levels and liver cancer risk

A total of 2 studies, published between 2017 and 2021, reported the association between serum HDL-C levels and liver cancer risk; these included 9,038,226 participants, among whom 27,657 cases of liver cancer were reported. Among these studies, 1 was conducted in Asia [28] and 1 was conducted in Europe [37]. A meta-analysis of the 2 studies showed no significant association between the serum HDL-C concentration and liver cancer risk (HR = 0.51, 95% CI = 0.23–1.13; *P* = 0.10). Significant heterogeneity was detected (I^2^ = 93%, *P* < 0.01) (Fig. 5). Due to the limited number of studies, Egger’s test and Begg’s test could not be performed. However, a visual inspection of the funnel plot did not reveal any asymmetry (Supplementary Fig S5).

### Sensitivity analysis and subgroup analysis

Sensitivity analysis was also conducted to explore the stability of the association between blood lipid levels and liver cancer risk in the included studies. After excluding the studies investigating LDL-C levels and liver cancer risk, the results remained consistent for TC, TG, and HDL-C levels when each individual study was removed.

We conducted several targeted subgroup analyses based on the characteristics of the studies to investigate the sources of heterogeneity. Due to insufficient research on the association between LDL-C levels and liver cancer risk, we only conducted subgroup analyses for TC, TG, and HDL-C levels. Regarding study quality, in high-quality studies, both TC (HR = 0.43, 95% CI = 0.33–0.55) and HDL-C levels (HR = 0.68, 95% CI = 0.47–0.99) were significantly associated with liver cancer risk, while there was no significant association between TG levels (HR = 0.83, 95% CI = 0.55–1.25) and liver cancer risk. According to the results of the subgroup analysis based on the geographic region of the study population, the associations between TC levels and liver cancer risk were similar in the European cohort (HR = 0.45, 95% CI = 0.35–0.59) and the Asian cohort (HR = 0.47, 95% CI = 0.33–0.69). In the Asian cohort, a significant association was observed between TG levels (HR = 0.59, 95% CI = 0.54–0.64) and liver cancer risk, while there was no association between HDL-C levels (HR = 0.75, 95% CI = 0.54–1.02) and liver cancer risk. In the European cohort, a significant association was also observed between HDL-C levels (HR = 0.68, 95% CI = 0.47–0.99) and liver cancer risk, but no significant association was found between TG levels (HR = 0.94, 95% CI = 0.58–1.53) and liver cancer risk. Additionally, in studies with a case number ≥ 266, TC levels (HR = 0.41, 95% CI = 0.31–0.54) were significantly correlated with liver cancer risk. Finally, we observed significant associations between TC (HR = 0.49, 95% CI = 0.37–0.65) and HDL-C levels (HR = 0.56, 95% CI = 0.43–0.72) and liver cancer risk in studies with a follow-up duration ≥ 10 years (Tables 1and Supplementary Table S4).

**Table 1:** Subgroup analysis of the association between total cholesterol levels and liver cancer risk

		Number of studies	HR (95% CI)	I^2^	P^a^	P^b^
Areas	Asian	4	0.47 (0.33, 0.69)	76.5%	< 0.01	0.14
	Europe	3	0.45 (0.35, 0.59)	78.1%	0.01	
Study quality	High quality	5	0.43 (0.33, 0.55)	79.3%	< 0.01	0.09
	Medium quality	2	0.61 (0.41, 0.90)	0.0%	0.39	
Follow -up years	≥ 10	5	0.49 (0.37, 0.65)	60.2%	0.03	0.16
	< 10	2	0.37 (0.35, 0.38)	0.0%	0.80	
Number of cases	≥ 266	4	0.41 (0.31, 0.54)	86.2%	< 0.01	0.20
	< 266	3	0.62 (0.44, 0.88)	0.0%	0.77	
Adjustment for confounders						
BMI	YES	5	0.46 (0.33, 0.64)	81.6%	< 0.01	0.18
	NO	2	0.46 (0.37, 0.57)	0.0%	0.826	
Alcohol drinking	YES	4	0.52 (0.36, 0.76)	83.5%	< 0.01	0.10
	NO	3	0.36 (0.22, 0.59)	63.7%	0.06	
Cigarette smoking	YES	5	0.46 (0.33, 0.64)	81.6%	< 0.01	0.18
	NO	2	0.46 (0.37, 0.57)	0.0%	0.826	
Physical activity	YES	3	0.50 (0.33, 0.77)	90.9%	< 0.01	0.10
	NO	4	0.43 (0.35, 0.52)	42.8%	0.136	
Dietary factors	YES	2	0.65 (0.45, 0.65)	0.0%	0.815	0.07
	NO	5	0.41 (0.32, 0.53)	81.6%	< 0.01	
Two aforementioned confounders	YES	6	0.46 (0.36, 0.59)	80.8%	< 0.01	0.23
	NO	1	0.41 (0.15, 1.12)	N/A	N/A	

## Discussion

This study conducted a comprehensive analysis by systematically integrating all relevant prospective research to further explore the association between blood lipid levels and liver cancer risk. The study included many participants from multiple countries, ensuring high reliability of the results. The cohort studies included in this analysis employed stringent participant selection criteria and excluded individuals diagnosed with liver cancer or other tumors during baseline measurements. This approach aimed to prevent interference from preexisting diagnoses of liver cancer or other tumors on the basis of variations in blood lipid concentrations. Consequently, the combined results of this meta-analysis hold substantial value in elucidating the causal relationship between blood lipid levels and the risk of liver cancer. The findings revealed that higher levels of TC, TGs, and HDL-C were associated with a reduced risk of liver cancer, whereas there was no correlation between serum LDL-C levels and liver cancer risk. These study results contribute to clarifying the link between blood lipid levels and liver cancer risk. Moreover, this discovery aids in the use of blood spectrum analysis to predict liver cancer risk in high-risk populations.

Metabolic alterations are widely recognized as hallmarks of cancer [39], and there is a close association between dyslipidemia and the development of liver cancer [23]. Previous research has indicated that elevated levels of TC increase the risk of cardiovascular diseases [40–42]. However, this study revealed a contrasting finding, indicating an actual association between higher TC levels and a reduced risk of liver cancer. The research findings remained robust during sensitivity analysis, and each subgroup analysis further confirmed this association. Due to the adverse impact of TC on the development of cardiovascular diseases, TC has been considered a substance that threatens human health. However, there is now a need to reevaluate the role of TC in the disease occurrence process. The serum TC concentration, which is commonly determined using a medical blood test, holds tremendous potential for reducing the incidence of liver cancer. This study included a significant number of participants and revealed a negative association between TC levels and the risk of liver cancer among both European and Asian populations in subgroup analyses. Regrettably, data regarding this association in other regions were not extracted in this study. Future prospective research involving participants from different regions would be beneficial for verifying the existence of this association, further substantiating and promoting the potential of measuring the serum TC concentration for preventing liver cancer. Additionally, factors such as smoking, alcohol consumption, and physical activity might exert varying influences on the association between serum TC levels and the risk of liver cancer. The impact of these potential confounding factors on the association between serum TC levels and the risk of liver cancer was largely excluded in most of the studies included in the analysis, while Lee et al. study did not thoroughly eliminate potential confounding factors, contributing to the discrepancy between their conclusions and the combined results of this study. In the liver, cholesterol is involved in bile acid synthesis, and bile acids have been shown to slow liver damage and the progression of HCC [43, 44]. Furthermore, studies suggest that receptor tyrosine kinases (RTKs) play a critical role in malignant tumor transformation and cancer metastasis [45] and that cholesterol inhibits RTK autophagic degradation in a GOLM1-dependent manner [46, 47]. Similarly, research has demonstrated that estrogen exerts a significant protective effect against HCC [48], and since cholesterol serves as a precursor for steroid hormones, epidemiological studies have shown an increased incidence of liver cancer in postmenopausal women [49]. However, further research is warranted to fully elucidate these potential mechanisms.

Previous studies have indicated an association between increased TG levels and an increasing risk of various cancers [41], such as colorectal cancer [50], prostate cancer [51], and breast cancer [52]. This meta-analysis combined the results of 8 prospective cohort studies and demonstrated a significant inverse association between TG levels and liver cancer risk. Notably, in the subgroup analyses of TC and HDL-C levels, an inverse association with the risk of liver cancer was observed in both subgroups with follow-up durations ≥ 10 years and < 10 years. However, regarding the TG subgroup analysis, a negative correlation with the risk of liver cancer was observed in the subgroup with a follow-up duration < 10 years, while no association was observed in the subgroup with a follow-up duration ≥ 10 years. Furthermore, in Asian populations, a negative correlation between TG levels and the risk of liver cancer existed, whereas no such association was observed in European populations. Thus, the association between TG levels and the risk of liver cancer may be influenced by the duration of follow-up and may also involve racial differences. The inconsistencies among the studies included in the analysis and the combined results could be attributed to variations in methodologies. For instance, the studies by Xia et al. [38] and Inoue et al. [29] categorized serum TG concentrations based on the critical value of normal TG levels, which might have limited the ability of these studies to observe the impact of higher or lower TG levels on the risk of liver cancer. Additionally, in the study by Nderitu et al. [37], non-fasting blood samples were collected from some participants during baseline lipid measurements, which might have led to variations in the serum TG concentration due to food intake prior to blood collection, resulting in contradictory findings compared to the pooled results. The potential mechanism underlying TGs as a protective factor against liver cancer in this study may be linked to the expression level of diacylglycerol acyltransferase (DGAT) [53], as confirmed in previous research. High DGAT2 expression is associated with prolonged overall survival in cancer patients [53, 54], and these findings have been validated via in vitro and in vivo experiments, indicating that DGAT2 overexpression can inhibit cell proliferation and reduce tumor growth [53]. DGAT is a key enzyme that facilitates the conversion of diacylglycerol (DAG) to triacylglycerol (TAG) [55], which is an essential step in fatty acid synthesis and storage. Normal TG metabolism is crucial for maintaining lipid balance in the liver and the whole body. Moreover, some studies have suggested that lower TG levels may be related to excessive fat accumulation in the liver and the development of fatty liver [56]. However, this remains a hypothesis, and further research is needed to explore the potential antitumor benefits of TGs.

Numerous studies have confirmed the potential protective role of HDL-C in the prevention of certain diseases [57], especially in the context of cardiovascular diseases [58]. A meta-analysis investigating the association between HDL-C levels and gastric cancer risk reported similar findings [59]. In the present study, a link was revealed between higher levels of HDL-C and a reduced risk of liver cancer. Among the studies included, Xia et al. [38] found no association between HDL-C levels and the risk of liver cancer, and Inoue et al. [29] observed no association between HDL-C levels and liver cancer risk only among females. This inconsistency with the combined results of this study might be because Xia et al. [38] and Inoue et al. [29] categorized their data based on normal HDL-C values, potentially overlooking the influence of other HDL-C values on the risk of liver cancer. Future research could further refine the measurement of HDL-C levels to derive more precise conclusions. However, the specific mechanisms underlying the role of HDL-C in reducing liver cancer risk remain unclear. First, HDL-C may act as a reverse transporter of cholesterol in the body, collecting excess cholesterol and facilitating its clearance [60, 61], thus maintaining cholesterol balance. Second, HDL-C also has antioxidant and anti-inflammatory properties, reducing intracellular oxidative stress and inflammation [62, 63], which are closely associated with the occurrence and development of cancer [64]. Reports have shown that HBV and HCV are independent risk factors for liver cancer [65, 66], and a case‒control study indicated lower levels of cholesterol, TGs, and HDL-C in the serum of HCV patients than in that of control individuals [67]. Although these observed phenomena are intriguing, direct evidence supporting the hypothesized negative association between HDL-C levels and liver cancer risk is currently lacking. Therefore, future research should further explore the physiological mechanisms underlying the association between HDL-C and liver cancer to better understand its role in the development of this disease.

In this meta-analysis, no significant association between LDL-C levels and liver cancer risk was observed. While other studies have suggested that lowering LDL-C levels can enhance the effectiveness of statin therapy for stroke patients [68] and that elevated LDL-C increases the risk of cardiovascular diseases [69, 70], similar results related to liver cancer were not observed in this study. Additionally, numerous studies have shown that high LDL-C levels are risk factors for various cancers, such as lung [71] and breast cancer [72]. However, the lack of association in this study may be attributed to the fact that elevated LDL-C levels are often associated with dyslipidemia [73], and the observed link between LDL-C levels and cancer risk might be a secondary effect. It is also possible that the impact of LDL-C on cancer risk varies depending on the cancer site. Furthermore, this study included only two prospective cohort studies, leading to a high level of heterogeneity (I^2^ = 93%). In the future, improvements in the study design and an increased sample size could be beneficial for obtaining a deeper understanding of the potential association between LDL-C levels and liver cancer risk.

Notably, among the commonly used drugs for controlling lipid levels, statins are increasingly associated with reducing the risk of various cancers, including liver cancer, as indicated by numerous studies [74, 75]. The primary mechanism of action of statins involves competitively inhibiting 3-hydroxy-3-methylglutaryl-coenzyme A reductase (HMGCR), thereby lowering lipid levels. Currently, the proposed mechanism by which statins prevent liver cancer involves modulating the tumor microenvironment and autophagic responses [76]. In this study, the reduction in lipid levels was linked to an increased risk of liver cancer, which seems contradictory to the protective role of statins against liver cancer. However, intriguingly, in Cho et al.‘s [28] study, the data from the subgroup analysis of individuals using statins still demonstrates an inverse association between lipid levels and the risk of liver cancer. The reasons for this discrepancy are not yet clear. However, it can be inferred that the mechanism underlying the negative association between lipid levels and the risk of liver cancer, which involves the use of statins to adjust the tumor microenvironment to prevent liver cancer, might not be significant. In other words, the protective effect of lipids against liver cancer might not be influenced by the use of statins. The protective mechanism of lipids against the risk of liver cancer might operate through the previously mentioned pathways, which requires further in-depth investigation. Additionally, dietary habits, particularly fat intake, are closely linked to lipid changes [77]. In recent years, several studies have confirmed the strong association between fat intake and the risk of liver cancer. An analysis by Salles et al. [78], based on data from the European Prospective Investigation into Cancer and Nutrition (EPIC) database involving 477,206 participants with a follow-up period of 11.4 years, revealed a negative association between total fat intake and the risk of liver cancer. Another longitudinal study indicated that the intake of plant-based fats is associated with a reduced risk of HCC [79]. The results of the present study indicate that lipids play a protective role against the risk of liver cancer. Considering the close association between fat intake and lipid levels, longitudinal studies on fat intake and the risk of liver cancer provide further supporting evidence for the findings of this study.

### Advantages and limitations

The strength of this meta-analysis lies in its comprehensive inclusion of large-scale, prospective studies with extended follow-up periods rather than reliance on case‒control studies, thus reducing potential limitations associated with case‒control designs that could affect the accuracy of the results. Additionally, the substantial number of participants and amount of case data ensures the validity and reliability of the findings. Furthermore, the results of sensitivity analyses were stable, and no evidence suggested publication bias in any of the included studies. Taken together, these factors contribute to enhancing the reliability of the research findings. Despite conducting numerous subgroup and sensitivity analyses, the statistical significance of the sources of heterogeneity in these variations was not definitively determined. However, the study results still demonstrated significant heterogeneity, which may impact the overall reliability of the pooled outcomes. This heterogeneity could stem from factors not accounted for in the analysis, such as lifestyle behaviors, individual differences, dietary habits, comorbidities, and psychological factors, which could influence disease incidence rates. Therefore, when interpreting the results, it is crucial to cautiously consider these potential confounding factors and comprehensively assess their potential impacts on the study outcomes. Additionally, while we employed explicit inclusion criteria, differences in comparison methods could lead to heterogeneity. For instance, comparing the highest reading of serum lipid profiles with the lowest reading may yield different estimates, as some studies may categorize groups based on quartiles of serum concentrations, while others may use normal ranges as the basis for categorization. As a result, to gain a more accurate understanding of the research findings, it is essential to integrate and fully comprehend the implications of these diverse measurement criteria on the study conclusions.

## Conclusion

Comprehensive analysis of the results revealed significant negative associations between liver cancer risk and serum levels of TC, TGs, and HDL-C. However, there is no significant association between serum LDL-C levels and liver cancer risk. Considering these findings, it is necessary to gain a thorough understanding of the potential underlying mechanisms involved. Moreover, further clinical research is needed to validate whether interventions to lower lipid concentrations can effectively reduce the risk of liver cancer.

### Supplementary Information

Below is the link to the electronic supplementary material.

Supplementary material 1 (DOCX 24.0 kb)

Supplementary material 2 (DOCX 15.4 kb)

Supplementary material 3 (DOCX 29.5 kb)

Supplementary material 4 (DOCX 23.7 kb)

Supplementary material 5 (ZIP 30.8 kb)

## Data Availability

All data and analysis processes have been presented in detail in this manuscript and the Supplementary Information section.
