# Supplementary material for: Association between blood lipid levels and the risk of liver cancer: a systematic review and meta-analysis
Source: Cancer Causes Control. 2024 Feb 20;35(6):943–53. doi: 10.1007/s10552-024-01853-9 (PMC11129988; doi:10.1007/s10552-024-01853-9)
Supplement: Supplementary file 2 — Supplementary material 2 (DOCX 15.4 kb) [file 10552_2024_1853_MOESM2_ESM.docx]

**Supplementary Table S2 Searching strategy for PubMed**

| #1 | **"Liver Neoplasms"[Mesh]** |
| --- | --- |
| #2 | **"neoplasms hepatic"[Title/Abstract] OR "neoplasms liver"[Title/Abstract] OR "liver neoplasm"[Title/Abstract] OR "neoplasm liver"[Title/Abstract] OR "hepatic neoplasms"[Title/Abstract] OR "hepatic neoplasm"[Title/Abstract] OR "neoplasm hepatic"[Title/Abstract] OR "cancer of liver"[Title/Abstract] OR "hepatocellular cancer"[Title/Abstract] OR "cancers hepatocellular"[Title/Abstract] OR "hepatocellular cancers"[Title/Abstract] OR "hepatic cancer"[Title/Abstract] OR "cancer hepatic"[Title/Abstract] OR "cancers hepatic"[Title/Abstract] OR "hepatic cancers"[Title/Abstract] OR "liver cancer"[Title/Abstract] OR "cancer liver"[Title/Abstract] OR "cancers liver"[Title/Abstract] OR "liver cancers"[Title/Abstract] OR "cancer of the liver"[Title/Abstract] OR "cancer hepatocellular"[Title/Abstract]** |
| #3＝#1 OR #2 | |
| #4 | “**Lipids/blood"[Mesh] OR "Triglycerides"[Mesh] OR "Cholesterol"[Mesh]** |
| #5 | "Triacylglycerol"[Title/Abstract] OR "Triacylglycerols"[Title/Abstract] OR "Epicholesterol"[Title/Abstract] OR "alpha Lipoprotein Cholesterol"[Title/Abstract] OR "Cholesterol alpha Lipoprotein"[Title/Abstract] OR "HDL Cholesterol"[Title/Abstract] OR "High Density Lipoprotein Cholesterol"[Title/Abstract] OR "Cholesterol HDL2"[Title/Abstract] OR "HDL2 Cholesterol"[Title/Abstract] OR "Cholesterol HDL3"[Title/Abstract] OR "HDL3 Cholesterol"[Title/Abstract] OR " Low Density Lipoprotein Cholesterol "[Title/Abstract] OR "beta Lipoprotein Cholesterol"[Title/Abstract] OR "Cholesterol beta Lipoprotein"[Title/Abstract] OR "LDL Cholesterol"[Title/Abstract] OR "Cholesteryl Linoleate LDL"[Title/Abstract] OR "LDL Cholesteryl Linoleate"[Title/Abstract] OR "lipids"[Title/Abstract] OR "serum lipid"[Title/Abstract] OR "Triglycerides"[Title/Abstract] OR "Triglyceride"[Title/Abstract] OR "Cholesterol"[Title/Abstract] OR "Total Cholesterol"[Title/Abstract] OR "TG"[Title/Abstract] OR "TC"[Title/Abstract] OR "HDLC"[Title/Abstract] OR "LDL-C"[Title/Abstract] |
| #6＝#4 OR #5 | |
| #7＝#3 AND #6 | |
