# Supplementary material for: Association between blood lipid levels and the risk of liver cancer: a systematic review and meta-analysis
Source: Cancer Causes Control. 2024 Feb 20;35(6):943–53. doi: 10.1007/s10552-024-01853-9 (PMC11129988; doi:10.1007/s10552-024-01853-9)
Supplement: Supplementary file 3 — Supplementary material 3 (DOCX 29.5 kb) [file 10552_2024_1853_MOESM3_ESM.docx]

**Supplementary Table S3. Characteristics of the included studies.**

| **First author**  **Publication year**  **Country**  （Cohort studies） | **Case/subject**  **Duration of follow-up（years）** | **Exposure categories** | **HR/RR（95%CI）** | **Adjustment for**  **potential confounding factors** | **NOS** |
| --- | --- | --- | --- | --- | --- |
| Sun 2021  China [32] | 298/114,972  10.86±2.11 years | Total： |  | age, gender, BMI, HDL-C, hs-CRP, TG, hypertension, diabetes, alcohol consumption, smoking physical activity and ALT | 9 |
|  |  | TC＜4.24 vs ≥4.24 mmol/L  Man:  TC＜4.24 vs ≥4.24 mmol/L  Woman:  TC＜4.24 vs ≥4.24 mmol/L | 0.58 (0.46,0.75)  0.57 (0.44,0.74)  0.56 (0.18,1.75) |  |  |
| Lee 2017  China [29] | 49/18,080  6.32years | Total： |  |  | 7 |
|  |  | Hypercholesterolemia vs non-hypercholesterolemia | 0.41 (0.15,1.11) |  |  |
| Cho 2021  Korea [26] | 26,891/ 8,528,790  7.3 years | Total： |  | age, sex, alcohol consumption, smoking history, regular physical activity, income, BMI, hypertension, DM, and current fenofibrate medication | 8 |
|  |  | TC 0–169 vs ≥215mg/dL | 0.36 (0.34,0.37) |  |  |
|  |  | TG 0–72 vs ≥158mg/dL  HDL 0–48 vs ≥67mg/dL  LDL 0–90 vs ≥132mg/dL | 0.59 (0.54,0.65)  0.87 (0.84,0.90)  0.35 (0.34,0.36) |  |  |
| Si 2016  Korea [31] | 36/3,544  1 years | Total： |  |  | 7 |
|  |  | TG≥150 vs < 150 mg/dL | 0.15（0.03,0.67） |  |  |
| Iso 2009  Japan [28] | 125/33,368  12.4 years | Man： |  | age, body mass index, pack year of smoking, ethanol intake, hypertension, diabetes, hyperlipidemia medication use, total vegetable intake, coffee intake and public health center. | 9 |
|  |  | TC4.65–5.16vs5.69–6.20(mmol/l) | 0.49（0.16 ,1.44） |  |  |
|  |  | Woman： |  |  |  |
|  |  | TC 4.65–5.16 vs 6.21+(mmol/l) | 0.80（0.28,2.27） |  |  |
| Borena 2012  Norway,Austria and Sweden [34] | 266/ 578,700  12 years | Total： |  | age,smoking,BMI | 8 |
|  |  | TC Q5 vs Q1 | 0.23（0.14,0.41） |  |  |
|  |  | TG Q5 vs Q1 | 0.59（0.24,1.43） |  |  |
| Nderitu 2017  Sweden [35] | 766/ 509,436  13 years | Total： |  | age (continuous), gender, SES, triglycerides (continuous), cholesterol (continuous), raised glucose, diabetic status and history of liver disease. | 9 |
|  |  | TC <4.70 vs ≥6.30 (mmol/L) | 0.46（0.37,0.57） |  |  |
|  |  | TG <0.7 vs ≥1.60(mmol/L)  HDL≥1.80 vs <1.25(mmol/L)  LDL≥4.22 vs <2.80(mmol/L) | 1.63（1.25,2.13）  0.42（0.26,0.68）  0.79（0.52,1.21） |  |  |
| Xia 2021  England, Wales, and Scotland [36] | 276/474,929  6.6 years | Man： |  | age in years, ethnic, education, index of multiple deprivation (a measure of socio-economic status), alcohol consumption, smoking status, physical activity, portions of fruit and vegetable intake, comorbidities, family history of cancer, menopause status (for the female only) hormone replacement therapy (for the female only) and levels of individual component of MetS | 8 |
|  |  | TG ≥1.7 vs <1.7 mmol | 0.87 (0.60,1.27) |  |  |
|  |  | HDL ≥1.03 vs <1.03mmol/L | 0.98 (0.67,1.43) |  |  |
|  |  | Woman： |  |  |  |
|  |  | TG ≥1.7 vs <1.7 mmol/L | 0.7 (0.43,1.14) |  |  |
|  |  | HDL ≥1.29 vs <1.29mmol/L | 0.82 (0.49,1.39) |  |  |
| Inoue 2009  Japan [27] | 114/27,724  10.2 years | Man： |  | age (stratified, 5-year age categories), study area (stratified, 10 public health center areas), smoking status (never, past, < 20 cigarettes per day, 20–29 cigarettes per day, Z 30 cigarettes per day), weekly ethanol intake ( < weekly, < 150 g per week, 150– < 300 g per week, Z 300 g per week), and total serum cholesterol (mg/dl, continuous) | 8 |
|  |  | TG≥1.69 vs 1.69mmol/l  HDL≥1.03 vs <1.03 mmol/L  Woman:  TG≥1.69 vs 1.69mmol/l  HDL ≥1.29 vs <1.29mmol/L | 0.98 (0.54,1.78)  0.44 (0.26,0.75)  0.58 (0.20,1.68)  1.19 (0.59,2.38) |  |  |
| Osaki 2012  Japan [30] | 129/ 23,625  9.1 years | Total： |  | age, smoking status (current smoker, ex-smoker), heavy drinking (more than 60 g pure alcohol), and every component of metabolic syndrome (high blood pressure, high triglycerides, low HDL, high glucose, and high BMI) | 7 |
|  |  | TG≥ 150 vs ＜150mg/dl | 0.52 (0.30,0.88) |  |  |
|  |  | HDL man≥40 vs < 40mg/dl and woman≥50 vs < 50mg/dl    Man: | 0.67 (0.45,0.99) |  |  |
|  |  | TG≥ 150 vs ＜150mg/dl | 0.73 (0.39,1.34) |  |  |
|  |  | HDL≥40 vs < 40mg/dl  Woman: | 0.66 (0.38,1.15) |  |  |
|  |  | TG≥ 150 vs ＜150mg/dl  HDL≥50 vs < 50mg/dl | 0.21 (0.06,0.70)  0.49 (0.27,0.89) |  |  |
| Chang 2022  China [25] | 228/52642  6 years | Total： |  | N/A | 7 |
|  |  | TG≥150 vs <150mg/dl | 0.22 (0.07,0.63) |  |  |
| Ahn 2009  Finland [33] | 191/29093  18 years | Total： |  | age, intervention, level of education, systolic blood pressure, body mass index, physical activity, duration of smoking, number of cigarettes smoked per day, saturates fat intake, polyunsaturated fat intake, total calorie, alcohol consumption, and serum HDL cholesterol. | 7 |
|  |  | TC≥276.7 vs <203.9mg/dl  HDL≥55.3 vs <36.2mg/dl | 0.66 (0.43,1.01)  0.61 (0.38,0.97) |  |  |

HR: Hazard Ratio, RR: Relative Ratio, BMI: Body Mass Index, N/A: Not Available.
