# Supplementary material for: Association between blood lipid levels and the risk of liver cancer: a systematic review and meta-analysis
Source: Cancer Causes Control. 2024 Feb 20;35(6):943–53. doi: 10.1007/s10552-024-01853-9 (PMC11129988; doi:10.1007/s10552-024-01853-9)
Supplement: Supplementary file 4 — Supplementary material 4 (DOCX 23.7 kb) [file 10552_2024_1853_MOESM4_ESM.docx]

Supplementary Table S4-1. Subgroup analysis of the association between triglyceride and liver cancer risk

|  |  | Number of studies | HR (95%CI) | I^2^ | P^a^ | P^b^ |
| --- | --- | --- | --- | --- | --- | --- |
| Areas | Asian | 5 | 0.59(0.54, 0.64) | 48.1% | 0.09 | 0.08 |
|  | Europe | 3 | 0.94(0.58, 1.53) | 79.8% | ＜0.01 |  |
| Study quality | High quality | 5 | 0.83(0.55, 1.25) | 88.8% | ＜0.01 | 0.37 |
|  | Medium quality | 3 | 0.39(0.25, 0.62) | 48.2% | 0.15 |  |
| Follow -up years | ≥10 | 3 | 0.99(0.58, 1.70) | 65.7% | 0.03 | 0.08 |
|  | ＜10 | 5 | 0.59(0.44, 0.78) | 56.1% | 0.04 |  |
| Number of cases | ≥266 | 4 | 0.83(0.51, 1.37) | 92.3% | ＜0.01 | 0.08 |
|  | ＜266 | 4 | 0.47(0.26, 0.85) | 58.0% | 0.05 |  |
| **Adjustment for confounders** | | | | | | |
| BMI | YES | 4 | 0.59(0.53, 0.64) | 12.3% | 0.33 | 0.29 |
|  | NO | 4 | 0.81(0.51, 1.29) | 78.8% | ＜0.01 |  |
| Alcohol drinking | YES | 6 | 0.60(0.55, 0.68) | 50.0% | 0.05 | 0.48 |
|  | NO | 2 | 1.08(0.40, 2.87) | 78.1% | 0.03 |  |
| Cigarette smoking | YES | 6 | 0.61(0.58, 0.66) | 32.8% | 0.17 | 0.30 |
|  | NO | 2 | 0.64(0.09, 4.59) | 92.3% | ＜0.01 |  |
| Physical activity | YES | 3 | 0.60(0.55, 0.66) | 60.5% | 0.06 | 0.22 |
|  | NO | 5 | 0.69(0.37, 1.26) | 82.2% | ＜0.01 |  |
| Dietary factors | YES | 1 | 0.80(0.60, 1.08) | 0.0% | 0.49 | 0.10 |
|  | NO | 7 | 0.73(0.50, 1.05) | 88.4% | ＜0.01 |  |
| Two aforementioned confounders | YES | 7 | 0.61(0.38, 0.99) | 86.3% | ＜0.01 | 0.19 |
|  | NO | 1 | 0.22(0.07, 0.63) | N/A | N/A |  |

Supplementary Table S4-1. Subgroup analysis of the association between high-density lipoprotein and liver cancer risk

|  |  | Number of studies | HR (95%CI) | I^2^ | P^a^ | P^b^ |
| --- | --- | --- | --- | --- | --- | --- |
| Areas | Asian | 3 | 0.75(0.54, 1.02) | 66.0% | 0.03 | 0.51 |
|  | Europe | 3 | 0.68(0.47, 0.99) | 62.9% | 0.04 |  |
| Study quality | High quality | 4 | 0.68(0.47, 0.99) | 69.2% | ＜0.01 | 0.44 |
|  | Medium quality | 2 | 0.65(0.48, 0.87) | 0.0% | 0.76 |  |
| Follow -up years | ≥10 | 3 | 0.56(0.43, 0.72) | 55.8% | 0.08 | 0.59 |
|  | ＜10 | 3 | 0.87(0.84, 0.90) | 0.0% | 0.55 |  |
| Number of cases | ≥266 | 3 | 0.77(0.58, 1.03) | 66.9% | 0.03 | 0.44 |
|  | ＜266 | 3 | 0.64(0.50, 0.82) | 40.5% | 0.17 |  |
| **Adjustment for confounders** | | | | | | |
| BMI | YES | 3 | 0.87(0.84, 0.90) | 48.0% | 0.15 | 0.43 |
|  | NO | 3 | 0.70(0.47, 1.05) | 69.0% | 0.01 |  |
| Alcohol drinking | YES | 5 | 0.87(0.84, 0.90) | 47.5% | 0.08 | 0.44 |
|  | NO | 1 | 0.42(0.26, 0.68) | N/A | N/A |  |
| Cigarette smoking | YES | 5 | 0.87(0.84, 0.90) | 47.5% | 0.08 | 0.44 |
|  | NO | 1 | 0.42(0.26, 0.68) | N/A | N/A |  |
| Physical activity | YES | 3 | 0.87(0.84, 0.90) | 0.0% | 0.45 | 0.37 |
|  | NO | 3 | 0.58(0.46, 0.75) | 59.4% | 0.06 |  |
| Dietary factors | YES | 2 | 0.82(0.63, 1.05) | 16.8% | 0.30 | 0.38 |
|  | NO | 4 | 0.67(0.47, 0.94) | 76.9% | ＜0.01 |  |
| Two aforementioned confounders | YES | 6 | 0.72(0.58, 0.90) | 64.9% | ＜0.01 | 0.64 |
|  | NO | 0 | N/A | N/A | N/A |  |
