# Supplementary figures and images for: Association between blood lipid levels and the risk of liver cancer: a systematic review and meta-analysis
Source: Cancer Causes Control. 2024 Feb 20;35(6):943–53. doi: 10.1007/s10552-024-01853-9 (PMC11129988; doi:10.1007/s10552-024-01853-9)

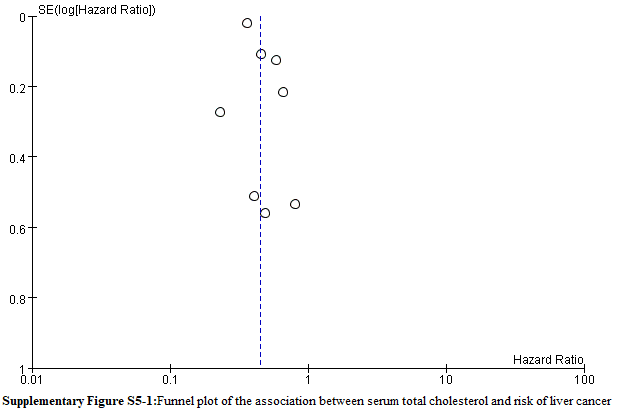

Supplement: Supplementary file 5 — Supplementary material 5 (ZIP 30.8 kb) [file 10552_2024_1853_MOESM5_ESM.zip › Supplementary Figure S5-1.png]

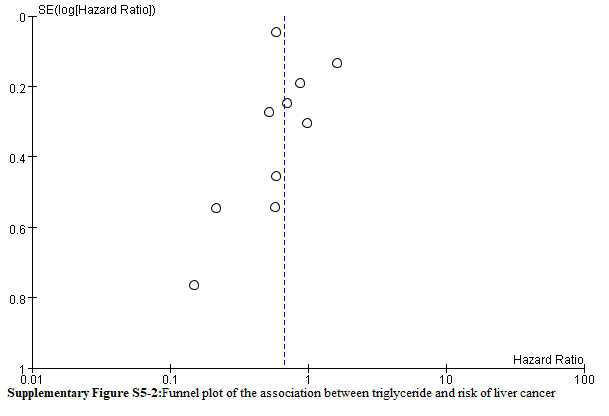

Supplement: Supplementary file 5 — Supplementary material 5 (ZIP 30.8 kb) [file 10552_2024_1853_MOESM5_ESM.zip › Supplementary Figure S5-2.png]

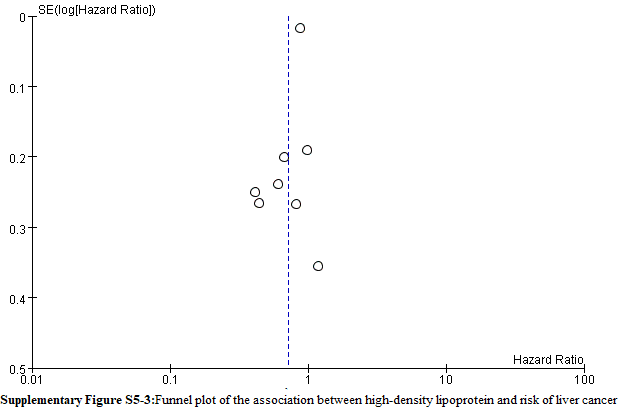

Supplement: Supplementary file 5 — Supplementary material 5 (ZIP 30.8 kb) [file 10552_2024_1853_MOESM5_ESM.zip › Supplementary Figure S5-3.png]

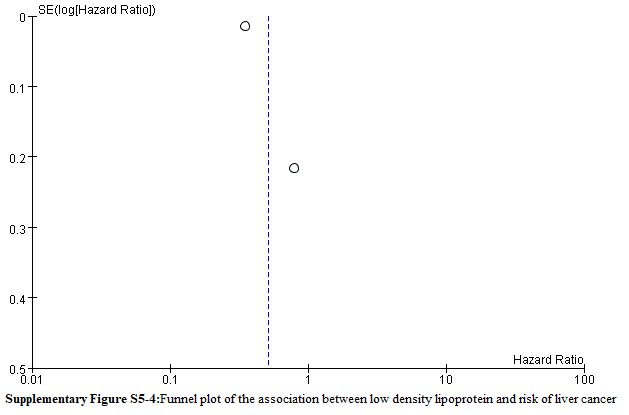

Supplement: Supplementary file 5 — Supplementary material 5 (ZIP 30.8 kb) [file 10552_2024_1853_MOESM5_ESM.zip › Supplementary Figure S5-4.png]
